# Senescent fibro-adipogenic progenitors are potential drivers of pathology in inclusion body myositis

**DOI:** 10.1007/s00401-023-02637-2

**Published:** 2023-09-29

**Authors:** Christopher Nelke, Christina B. Schroeter, Lukas Theissen, Corinna Preusse, Marc Pawlitzki, Saskia Räuber, Vera Dobelmann, Derya Cengiz, Felix Kleefeld, Andreas Roos, Benedikt Schoser, Anna Brunn, Eva Neuen-Jacob, Jana Zschüntzsch, Sven G. Meuth, Werner Stenzel, Tobias Ruck

**Affiliations:** 1 Department of Neurology, Medical Faculty, Heinrich Heine University Duesseldorf, Moorenstr. 5, 40225 Duesseldorf, Germany; 2 Department of Neuropathology, Charité–University Medicine Berlin, Bonhoefferweg 3, 10117 Berlin, Germany; 3 Department of Neurology, Charité–University Medicine Berlin, Bonhoefferweg 3, 10117 Berlin, Germany; 4 Department of Neuropediatrics, Developmental Neurology and Social Pediatrics, Centre for Neuromuscular Disorders in Children, University Hospital Essen, University of Duisburg-Essen, Essen, Germany; 5 Friedrich Baur Institute at the Department of Neurology, LMU University Hospital, LMU Munich, 80336 Munich, Germany; 6 Institute of Neuropathology, Heinrich Heine University, University Hospital of Düsseldorf, Düsseldorf, Germany; 7 Department of Neurology, University Medical Center Göttingen, Robert-Koch-Str. 40, Göttingen, Germany

**Keywords:** Senescence, Inclusion body myositis, Acetylcholine receptor, Single nuclei, Myofiber, Complement

## Abstract

Inclusion body myositis (IBM) is unique across the spectrum of idiopathic inflammatory myopathies (IIM) due to its distinct clinical presentation and refractoriness to current treatment approaches. One explanation for this resistance may be the engagement of cell-autonomous mechanisms that sustain or promote disease progression of IBM independent of inflammatory activity. In this study, we focused on senescence of tissue-resident cells as potential driver of disease. For this purpose, we compared IBM patients to non-diseased controls and immune-mediated necrotizing myopathy patients. Histopathological analysis suggested that cellular senescence is a prominent feature of IBM, primarily affecting non-myogenic cells. In-depth analysis by single nuclei RNA sequencing allowed for the deconvolution and study of muscle-resident cell populations. Among these, we identified a specific cluster of fibro-adipogenic progenitors (FAPs) that demonstrated key hallmarks of senescence, including a pro-inflammatory secretome, expression of p21, increased β-galactosidase activity, and engagement of senescence pathways. FAP function is required for muscle cell health with changes to their phenotype potentially proving detrimental. In this respect, the transcriptomic landscape of IBM was also characterized by changes to the myogenic compartment demonstrating a pronounced loss of type 2A myofibers and a rarefication of acetylcholine receptor expressing myofibers. IBM muscle cells also engaged a specific pro-inflammatory phenotype defined by intracellular complement activity and the expression of immunogenic surface molecules. Skeletal muscle cell dysfunction may be linked to FAP senescence by a change in the collagen composition of the latter. Senescent FAPs lose collagen type XV expression, which is required to support myofibers’ structural integrity and neuromuscular junction formation in vitro. Taken together, this study demonstrates an altered phenotypical landscape of muscle-resident cells and that FAPs, and not myofibers, are the primary senescent cell type in IBM.

**Supplementary Information:**

The online version contains supplementary material available at 10.1007/s00401-023-02637-2.

## Introduction

The spectrum of idiopathic inflammatory myopathies (IIM) is characterized by muscle inflammation as a pathogenic hallmark [1, 36]. Among IIM, inclusion body myositis (IBM) is a unique entity due to its characteristic clinical presentation, advanced age at onset, and notable refractoriness to contemporary treatment strategies [17]. As such, IBM leads to progressive muscle damage and weakness resulting in an unmet need for novel treatment approaches.

One explanation for the treatment refractoriness of IBM might be the instigation of cell-autonomous mechanisms that promote disease progression independent of ongoing inflammatory activity. Skeletal muscle is constituted of myogenic and non-myogenic cells. The complex interplay of these cells is required to maintain muscle health and for the resolution of inflammation [39, 43]. However, IBM imposes a burden of chronic inflammation on the compartment, potentially shifting the cellular homeostasis to a detrimental muscle phenotype [7, 17, 40]. Indeed, in response to chronic stress or damage, cells may assume a state of stable cell cycle arrest termed cellular senescence [44]. These cells continue to influence their environment by engaging a specific senescence-associated secretory phenotype (SASP) characterized by immunomodulatory cytokines, growth factors, and proteases. Following this line of argumentation, accumulation of senescent cells in skeletal muscle may constitute a cell-autonomous mechanism by which muscle-resident cells promote compartmentalized inflammation and fibrotic remodelling in IBM.

Succeeding the identification and characterization of highly cytotoxic, terminally differentiated CD8 T cells [16, 18], renewed attention has been focused on the development of immunomodulatory strategies for IBM, e.g. the depletion of KLRG1^+^ CD8 T cells [14]. However, addressing the deterioration of the skeletal muscle compartment might improve efficacy of those treatment strategies.

To understand whether IBM instigates cell-autonomous mechanisms, we studied cellular senescence in the skeletal muscles of IBM patients and compared them with those of non-diseased controls (NDC) and immune-mediated necrotizing myopathies (IMNM) as diseased control. Employing a single-nuclei transcriptomic approach, we describe a novel population of senescent fibro-adipogenic progenitors (FAPs) that may induce distinct alterations of the myogenic compartment. Given the growing interest and availability of senotherapeutics [21, 44], targeting these cells may provide an innovative strategy to ameliorate the muscle phenotype of IBM.

## Methods

### Ethics statement

The study was conducted in accordance with the Declaration of Helsinki and approved by the ethics committee of the Heinrich Heine University Duesseldorf (2016-053-f-S and 2021-1417) and the Charité Berlin (EA2/163/17). This study analysed skeletal muscle biopsies acquired from IIM patients and non-diseased controls (NDCs). All patients signed written informed consent before acquisition of the biopsy and clinical metadata.

### Patient recruitment and clinical data

Patients were recruited from three tertiary centres specialized in the management of IIM (University Hospital Duesseldorf, Charité–University Medicine Berlin, and University Medical Center Göttingen). Patients treated in the outpatient clinic and patients admitted to the hospital were asked for study inclusion as well as written consent. Patients were recruited from January 2014 to January 2022. As disease control, we chose immune-mediated necrotizing myopathy (IMNM) as these patients rarely demonstrate extra-muscular manifestations, similar to IBM. IBM and IMNM patients were required to meet the European Neuromuscular Centre (ENMC) criteria for diagnosis [1, 2, 54]. NDCs served as an additional control cohort. As previously proposed by our group [53], these patients underwent muscle biopsy for diagnostic purposes, e.g. for myalgia. These patients were required not to have any objective muscle weakness or abnormal creatine kinase levels. On muscle histology, the patient's specimens were required to demonstrate no signs of inflammation or any other structural abnormalities. These patients had no myositis-specific or myositis-associated antibodies. Given the impact of age on the study readout of cellular senescence, NDC and IMNM patients were age-matched to IBM patients (maximum difference in age of 3 years). This study included 16 IBM, 16 NDC and 16 IMNM patients. The individual number of patients is given for each experiment as indicated. The disease duration was defined as the time between the first symptoms as reported by the patients to the time of muscle biopsy.

### Biomaterial

All skeletal muscle specimens had been cryopreserved at − 80 °C before analysis according to the predefined standard operating procedure at the local biobank of the Heinrich Heine University Duesseldorf and the Charité Berlin.

### Immunofluorescence

Immunofluorescence (IF) was performed as previously described [53]. Briefly, all stains were performed on 8 µm cryostat sections. We used irrelevant antibody stains (either mouse/rabbit monoclonal/polyclonal isotype controls) as negative controls, as well as omission of the primary antibody. The following antibodies were used for staining procedures: Laminin-β1 (Rabbit, 1:100, Novus Biologicals), p21 (Mouse, 1:50, Novus Biologicals), C3 (Rat, 1:100, Santa Cruz), PDGFRa (Mouse, 1:50, Novus Biologicals), α-bungarotoxin (Snake, 1:50, Invitrogen). The secondary antibodies for immunofluorescence were anti-rabbit (Alpaca, 1:200, Jackson ImmunoResearch), anti-rat (Alpaca, 1:200, Jackson ImmunoResearch) and anti-mouse (Alpaca, Jackson ImmunoResearch). Specimen were analysed using Zeiss Axio (for 10 and 20-fold magnification) or LSM 880, Zeiss (for 40 and 63-fold magnification) in cooperation with the Core Facility for Advanced Light Microscopy, Heinrich Heine University Düsseldorf. The biopsies were blinded for quantification with the diagnosis not possible to identify from the label. The scoring was performed in randomly distributed 10 high-power fields (HPF, based on the microscope used and the respective oculars ≙ 0.16 mm^2^) as previously described [53].

### Quantitative reverse transcription PCR (qRT-PCR)

Total RNA was extracted from muscle specimens as previously described [53]. cDNA was synthesized using the High-Capacity cDNA Archive Kit (Applied Biosystems, Foster City, CA). For reactions, 10 ng of cDNA were used on the 7900HT Fast Real-Time PCR System (Applied Biosystems, Foster City, CA) with the following running conditions: 95 °C 0:20, 95 °C 0:01, 60 °C 0:20, 45 cycles (values above 40 cycles were defined as “not expressed”). All targeted transcripts were run as triplicates. For each of these runs, the reference gene *PGK1* has been included as internal control to normalize the relative expression of the targeted transcripts. The 2^−ΔΔCT^ method was used to quantify gene expression of IBM and IMNM patients compared to NDCs.

### Isolation, purification, and cultivation of primary human muscle cells

Muscle tissue was dissociated using a muscle dissociation kit (Miltenyi Biotec, Bergisch Gladbach, Germany) according to the manufacturer’s instructions. For primary human skeletal muscle cell (PHMC) purification, we used a CD56 (clone N901, Beckman Coulter) antibody combined with microbead magnetic separation. For cultivation, we used Biolaminin 521 LN coated 6-well plates and skeletal muscle cell growth medium (PELOBiotech, Planegg, Germany) according to the manufacturer's instructions. For all experiments, we used differentiated PHMCs by allowing cells to grow to 100% confluence. These PHMC cultures contain different degrees of myoblast differentiation as well as myotubes.

### Flow cytometry

PHMC were analysed by flow cytometry. Cells were treated and cultivated as indicated in the corresponding section. Cells were then washed with ice-cold PBS and scraped. The cells were centrifuged, and the pellet was washed before staining. PHMC were stained with the α-bungarotoxin antibody (CF555, Invitrogen). 1 × 106 PHMC per well was co-cultivated with the indicated concentrations of collagen for 24 h. Recombinant COL1A1 and COL15A1 were purchased from Abbexa, Cambridge, England.

### Live/dead assay

For the assessment of viability, we used the LIVE/DEAD Viability/Cytotoxicity Kit for mammalian cells (Invitrogen, Waltham, Massachusetts) according to the manufacturer's instructions. Briefly, 1 × 10^6^ PHMC per well were co-cultivated with the indicated concentrations of collagen for 24 h. Cell viability was assessed by fluorescence microscopy. Live cells were identified by green-fluorescent calcein-AM indicating intracellular esterase activity. Dead cells were identified by red-fluorescent ethidium homodimer-1 indicating a loss of membrane integrity. For each sample, 100 cells were counted and the frequencies of live or dead cells were recorded.

### ATPase staining

ATPase enzyme histochemical preparations at pH 4.3, 4.6, and 9.4 were performed according to standard protocols to highlight type 1 and type 2A and 2X fibers [10].

### Isolation of single nuclei from muscle biopsies

Single nuclei were isolated from frozen muscle biopsy specimens. Approximately 60 mg of muscle was used for each sample. All biopsies were taken from the quadriceps muscle (vastus medialis) approximately 3 cm proximal to the knee joint.

The single nuclei suspension was obtained using GEXSCOPE^®^ Single Nucleus RNA Library Kit V2 (Singleron Biotechnologies) as previously described [34]. Briefly, on ice, the tissue was immersed in a cold nucleus separation solution and cut into small pieces. Further homogenization was achieved by performing 5 strokes with pestle A and 5 strokes with pestle B of the Kimble douncer (KIMBLE^®^ KONTES^®^ Dounce Tissue Grinder). The sample was then incubated on ice for 15 min, where the state of dissociation was monitored every 5 min under a light microscope. Following homogenization and digestion, the suspension was filtered using a 40 µm sterile strainer. The nuclei suspension was centrifuged at 200×*g* for 2 min at 4 °C, and the supernatant was centrifuged at 500×*g* for 5 min at 4 °C. The resulting pellet containing nuclei was resuspended in 0.25 ml cold nuclei suspension buffer. The quality of the nuclei was assessed by Trypan Blue staining (0.4% w/v, Gibco) under a light microscope. The nuclei were counted using propidium iodine with a Luna FX7 automated cell counter (Logos Biosystems, Villeneuve d’Ascq, France).

### Library generation and sequencing

A total of 30 000 nuclei were loaded onto a microfluidic chip (Singleron GEXSCOPE^®^ Single Nucleus RNA Library Kit V2) for a minimal capture of 6000 nuclei for each sample. Barcoded beads containing a unique cell barcode were loaded into the chip, and nuclei were lysed. After nuclei lysis, polyadenylated RNA was captured onto the Barcode Beads by the poly (dT) sequence. Barcode Beads with captured mRNA molecules were collected and subjected to reverse transcription reaction. The cDNA was then amplified and QC. NGS libraries generated were sequenced on an Illumina NovaSeq 6000 instrument using a paired-end 150 bp approach. The reads were demultiplexed on Illumina’s BaseCloud, and fastq files were used to initiate data analysis.

### Bioinformatics workflow

Illumina reads (fastq files) were processed to gene expression matrices using CeleScope^™^ (v1.14.1., www.github.com/singleron-RD/CeleScope; Singleron Biotechnologies). Briefly, fastq files were demultiplexed according to their respective cell barcodes and UMIs. Adapter sequences and poly-A tails were trimmed (cutadapt; https://cutadapt.readthedocs.io/en/stable/installation.html) and the trimmed Read2 reads were aligned to the GRCh38 version of the human genome with Ensembl version 92 gene annotations (STAR v2.6.1a_08-27 [https://github.com/alexdobin/STAR] and featureCounts 2.0.1). We excluded cells with a unique feature count over 2500 or below 200 or cells with a mitochondrial ratio of more than 5%. A combined digital expression matrix was constructed, containing all sequenced experiments, for downstream analysis. Analysis was performed in Seurat (v4.3.0). Data were normalized using the NormalizeData function and 2000 features with high cell-to-cell variation were calculated using the FindVariableFeatures function. For data integration of IBM and NDC samples, we employed the integration workflow as proposed for the Seurat workspace [57]. The FindIntegrationAnchors function was used to assemble all snRNA-seq datasets into an integrated and unbatched dataset. Next, to reduce dimensionality of the datasets, the RunPCA function was conducted with default parameters on linear-transformation scaled data generated by the ScaleData function. After integration, we performed a modularity-optimized Louvain clustering with a resolution of 0.8.

### Cluster annotations

The FindAllMarkers function was used to identify cluster-specific markers with a fold-change threshold of 0.25 and min.pct set to 0.25. Clusters were then classified and annotated based on expressions of canonical markers of particular cell types. The complete list of markers for all clusters is available as Supplemental File 1.

### Subclustering

As indicated, clusters of interest were extracted from the integrated dataset for in-depth analysis and subclustering. Principal component analysis and clustering were repeated as described above.

### Differential expression testing and functional enrichment analysis

Differential gene expression testing was performed using the FindMarkers function using Wilcox testing and a fold-change threshold of 0.25. The Bonferroni correction was used based on the total number of genes in the dataset. Differentially expressed genes (DEGs) were selected based on the adjusted *p* value.

DEGs were used for gene set enrichment analysis (GSEA) using the ClusterProfiler package (v4.3.1) and the Gene Ontology (GO) “Biological Process” and the “Reactome” databases.

For targeted GSEA analysis, we used Genetrail (https://genetrail.bioinf.uni-sb.de/start.html) with the SenMayo gene set (v2023.1.Hs, available from https://www.gsea-msigdb.org/gsea/index.jsp as “SAUL_SEN_MAYO”).

Cell–cell communication was analysed using the CellChat package (v1.5.0) as proposed by the developers.

### Statistical analysis

Statistical analysis was performed using *R* 3.5.3. Data were presented as median with IQR, mean ± standard deviation (SD), as absolute (*n*) or relative frequencies (%). Differences between the two groups were analysed using the Mann–Whitney *U* test. The Kruskal–Wallis test was used for multiple groups.

*p* > 0.05 was classified as not significant, *p* < 0.05 as significant (*), *p* < 0.01 (**), *p* < 0.001 (***).

## Results

### p21^+^ senescent cells are abundant in the muscle of IBM

First, we sought to determine whether the number of senescent cells is altered in IBM compared to NDC and IMNM patients. For this purpose, we recruited a multicentric cohort of 48 patients (16 per group) in three tertiary centres specialized in managing IIMs. The clinical and epidemiological characteristics are given in Table 1. Given that age is likely to have a major impact on the development of cellular senescence, NDC and IMNM patients were matched to IBM by age to account for this confounder. For diagnosis of IBM and IMNM, patients were required to meet the current ENMC criteria for diagnosis, respectively [2, 35]. Briefly, IBM patients were 73 (45–87) years old with 7 males and 9 females. 5 out of 16 patients received intravenous immunoglobulin treatment at the time of biopsy. No other immunosuppressants were recorded for the IBM cohort.

**Table 1:** Clinical and demographic characteristics

Characteristics	IBM (*n* = 16)	NDC (*n* = 16)	IMNM (*n* = 16)
Median age at diagnosis (years, range)	73 (45–87)	72 (44–86)	74 (46–85)
Median disease duration* (months, range)	7 (0–32)	3 (0–18)	2 (1–6)
Gender, *n* (%)			
Female	9 (56%)	8 (50%)	7 (44%)
Male	7 (44%)	8 (50%)	9 (56%)
Creatine kinase (U/I, mean, SD)	209 (45)	104 (98)	1509 (540)
Antibody status, *n* (%)			
Anti-cN-1A	9 (56%)	0 (0%)	0 (0%)
Anti-SRP	0 (0%)	0 (0%)	7 (44%)
Anti-HMGCR	0 (0%)	0 (0%)	8 (50%)
None	7 (44%)	16 (100%)	1 (6%)
Symptoms, *n* (%)			
Muscle weakness and atrophy	16 (100%)	0 (0%)	15 (94%)
Myalgia	2 (13%)	11 (69%)	11 (69%)
Dysphagia	12 (76%)	2 (13%)	7 (44%)
Lung involvement	0 (0%)	0 (0%)	4 (25%)
Treatment, *n* (%)			
Treatment naïve	11 (69%)	15 (94%)	2 (13%)
IVIG	5 (31%)	0 (0%)	4 (25%)
Steroids	2 (13%)	1 (6%)**	9 (56%)
Steroid dose per day (mean, SD)	10 (10)	5 (0)	30 (25)
Azathioprine	0 (0%)	0 (0%)	2 (13%)
Rituximab	0 (0%)	0 (0%)	2 (13%)

*HMGCR* HMG-CoA reductase; *IBM* inclusion body myositis; *IMNM* immune-mediated necrotizing myopathy; *NDC* non-diseased control; *SD* standard deviation; *SRP* signal recognition particle

*Disease duration was defined between the time of first symptoms as reported by the patient and the time of biopsy

**One NDC received steroids for the treatment of psoriasis

**Fig. 1:**
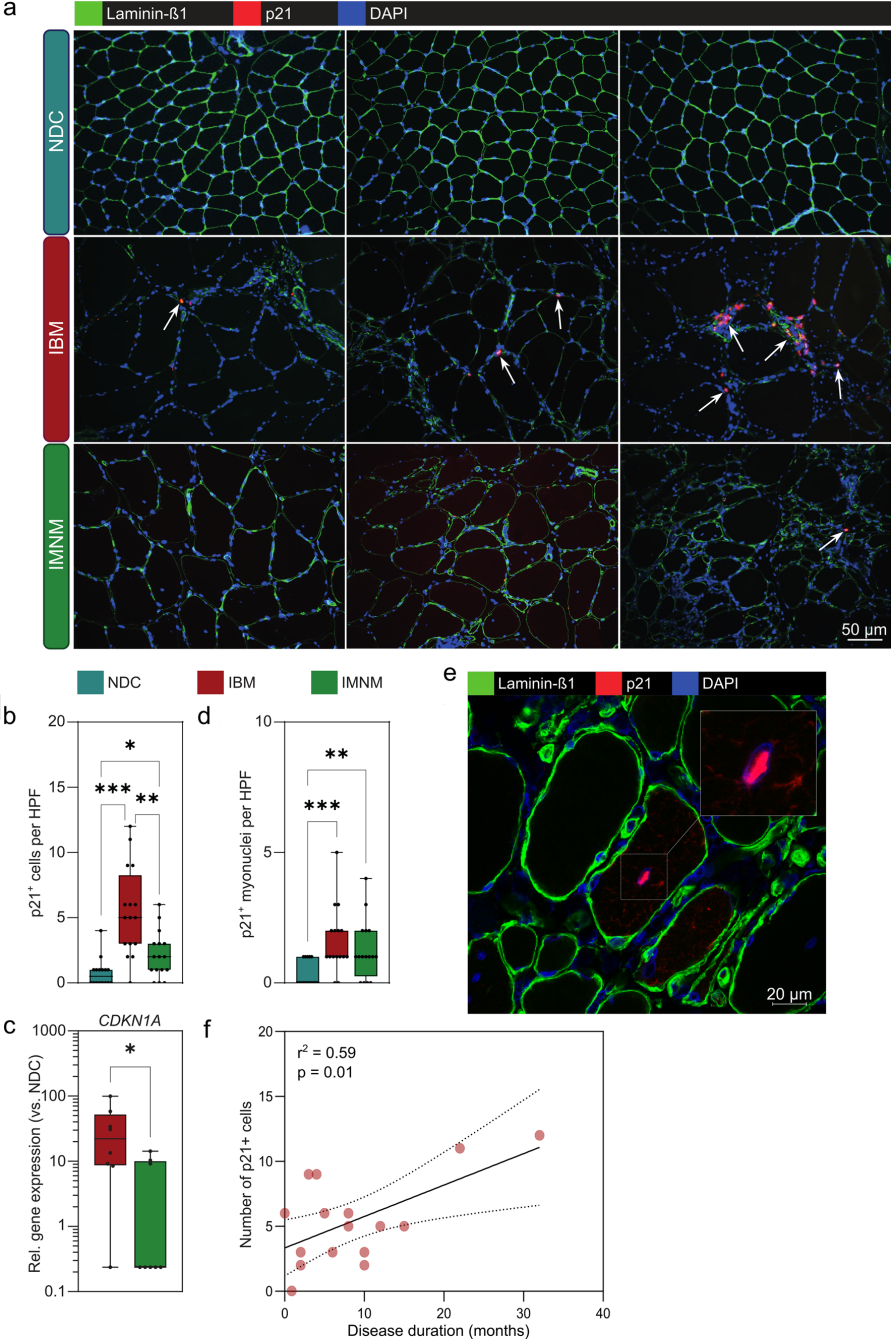
p21^+^ senescent cells are abundant in muscle of IBM. **a** Immunofluorescence staining of p21 (red), laminin-β1 (green), and DAPI (blue) in muscle specimens of NDCs (*n* = 16), IBM (*n* = 16), and IMNM (*n* = 16) patients. Patients were matched by age. Arrows indicate single p21^+^ cells or clusters of p21^+^ cells. **b** p21 + cells were counted in randomly distributed 10 HPF (≙ 0.16 mm^2^). The biopsies were blinded for quantification, with the diagnosis impossible to identify from the label. p21^+^ cells were defined as cells with a clear expression of p21 in the nucleus. **c** RT-qPCR analysis of *CDKN1A* coding for p21 in muscle specimen (*n* = 16 per group). The 2^−ΔΔCT^ method was used for normalization. CDKN1A is significantly over-expressed to NDC for both IBM and IMNM. **d** Quantification of p21^+^ myonuclei in muscle in muscle specimen (*n* = 16 per group). p21^+^ myonuclei were defined as nuclei located inside a muscle fiber. **e** Exemplary image of a p21+ nucleus located inside a myofiber. Differences between groups were analysed by Kruskal–Wallis test followed by post hoc testing. **f** Simple linear regression of the number of p21+ cells and the disease duration of each IBM patient. The disease duration was defined as the time in months between the first symptoms as reported by the patient and the time of biopsy. The dotted line indicates the 95% confidence interval. Significance was tested by the likelihood test. *p < 0.05, **p < 0.01, ***p < 0.001. *NDC* non-diseased control; *HPF* high-power field; *IBM* inclusion body myositis; *IMNM* immune-mediated necrotizing myopathy; *r2* coefficient of determination; *RT-qPCR* real-time quantitative polymerase chain reaction

As the first readout, we focused on the study of p21, an established marker for cellular senescence [15]. This choice was motivated by several recent studies in murine and human tissues demonstrating that p21 governs a senescent program in skeletal muscle and serves as a marker for muscle cell senescence [12, 50, 64]. We assessed the number of p21^+^ cells by IF (Fig. 1a). Given the nuclear expression of p21, only cells that demonstrated clear co-localization of p21 and DAPI were considered to be p21^+^. Indeed, the number of p21^+^ cells was higher in IBM as compared to NDC and IMNM patients (Fig. 1b). To further corroborate this data, we also analysed p21 at the gene level by PCR observing an increased expression of *CDKN1A* (coding for p21) in IBM compared to NDC or IMNM patients (Fig. 1c). Intriguingly, a substantial number of p21^+^ cells were detected in the perimysial area in IBM. To clarify the origin of those p21^+^ cells, we also counted the number of p21^+^ myonuclei (Fig. 1d). Myonuclei were defined as nuclei localized inside the boundaries of the corresponding myofiber (Fig. 1e). IBM and IMNM patients demonstrated similar numbers of p21^+^ myonuclei leading us to hypothesize that non-myogenic cells resident in skeletal muscle might primarily assume a senescent phenotype in IBM. Of note, longer disease durations were associated with an increased number of p21^+^ cells (Fig. 1f). Following this line of argumentation, we aimed to study senescence at the level of single cells (or nuclei) in the landscape of myogenic and non-myogenic cells in the IBM muscle.

### Single-nuclei RNA sequencing deconvolutes distinct cell populations and allocates senescence in IBM muscle mainly to fibro-adipogenic progenitors

For this purpose, we performed snRNA-seq of three IBM patients and three NDCs. We chose snRNA-seq as an alternative to single-cell RNA-seq due to the large size of myofibers that restricts the latter’s use for studying skeletal muscle [30, 50]. For snRNA-seq, all muscle specimens were obtained from the quadriceps muscle, specifically the vastus medialis, approximately 3 cm proximal to the knee joint. IBM patients were 65, 68, and 71 years old with two male and one female subject. NDCs were age- and sex-matched. All IBM patients and all NDCs were treatment naïve.

**Fig. 2:**
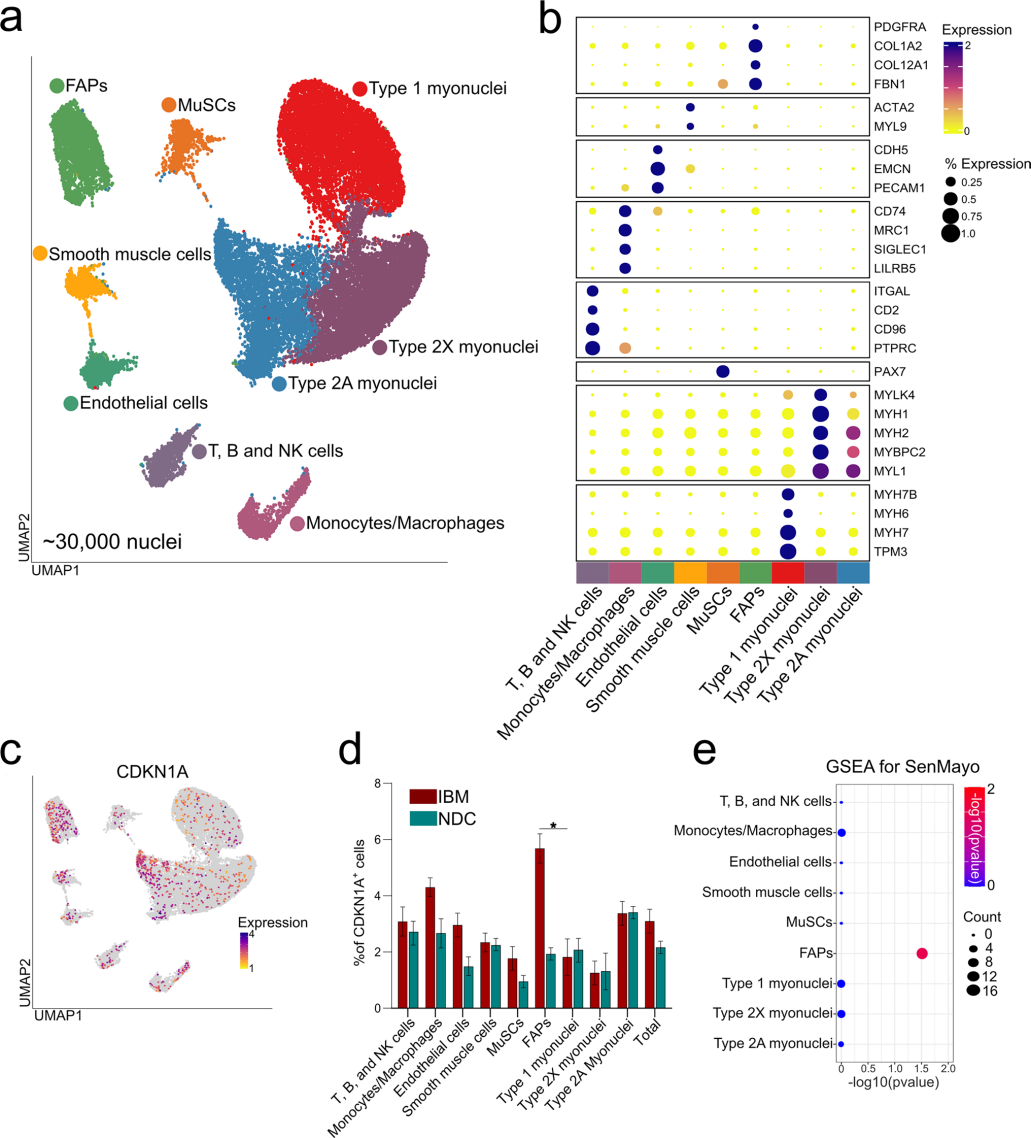
Single-nuclei RNA sequencing of IBM and NDC muscle. A total of 6 frozen muscle specimens were processed for single nuclei-RNAseq (3 samples per group). A total of ~ 30,000 nuclei were included for downstream processing and clustering after quality control. **a** UMAP embedding demonstrating distinct clusters of cell types and subtypes. **b** Clustered dot plot visualization of top-regulated marker genes. The mean expression for each cluster is indicated by colour code. The dot size indicates the percent of expressing cells. Clusters were annotated based on marker genes. **c** Expression of CKDN1A (coding for p21) across the UMAP embedding. The mean expression for each cell is indicated by the colour code. **d** Frequency of CDKN1A+ cells for each cell cluster as indicated for IBM patients and NDC. Differences between groups were analysed by the Kruskal–Wallis test followed by post hoc testing. **e** Gene set enrichment analysis (GSEA) for the SenMayo dataset. Differentially expressed genes were determined by the FindMarkers function using Wilcox testing and a fold-change threshold of 0.25. The Bonferroni correction was used for correction for multiple testing. DEGs specific to the IBM dataset were entered into the GSEA. The Kolmogorov–Smirnov test, followed by post hoc correction, was used to determine the significance. **p < 0.01. *ACTA2* actin alpha 2; *CDKN1A* cyclin dependent kinase inhibitor 1A; *CDH5* cadherin 5; *COL* collagen; *EMCN* endomucin; *FBN1* fibrillin-1; *ITGAL* integrin subunit alpha L; *LILRB5* leukocyte immunoglobulin like receptor B5; *MRC1* mannose receptor C-type 1; *MYBPC2* myosin binding protein C2; *MYL9* myosin light chain 9; *NDC* non-diseased control; *IBM* inclusion body myositis; *SIGLEC1* sialic acid binding Ig like lectin 1; *TPM3* tropomyosin 3; *PECAM1* platelet and endothelial cell adhesion molecule 1; *PAX7* paired box 7; *PTPRC* protein tyrosine phosphatase type C (CD45); *UMAP* uniform manifold approximation and projection

After quality control, ~ 30,000 nuclei of which ~ 13,000 were obtained from IBM muscle and ~ 17,000 from NDC were integrated into a batch-corrected expression matrix for downstream analysis. Using graph-based clustering of uniform manifold approximation and projection (UMAP), we observed nine major cell types or subtypes based on differential marker expression (Fig. 2a, b). Cell types were assigned according to the expression of canonical markers (a full list of all marker genes is given in Supplemental File 1) based on previous transcriptomic studies of skeletal muscle [9, 30, 50, 52]. While non-myogenic cells were separated based on their transcriptomic profiles, the classification of myogenic cells was more challenging. Briefly, human skeletal muscle may be classified into slow-twitch (type 1) and fast-twitch (type 2A and type 2X) myofibers based on the presence or absence of specific myosin heavy chains (MYH) [42]. For this study, we annotated the myogenic compartment based on this classification, although it should be noted that some myofibers are considered hybrids falling between canonical subtypes [58]. First, we annotated type 1 myonuclei based on the expression of canonical markers such as *MYH7* or *MYH7B* (Fig. 2a, b) [50, 58]. Second, a subgroup of fast-twitch (type 2X) myonuclei were identified based on the expression of *MYH1* and the absence of *MYH7* [50, 58]. As previously described by Murgia et al., specific markers, except *MYH2*, for the type 2A subgroup of fast-twitch myofibers are currently lacking [42]. Following this line of argumentation, we annotated the final myonuclei cluster as type 2A given the absence of *MYH7* and *MYH1* expression specific for type 1 and type 2X fibers, respectively.

To understand the distribution of senescence in myogenic and non-myogenic cells, we determined the expression of *CDKN1A* across the dataset (Fig. 2c). To compare the cluster-specific frequencies, we calculated the number of *CDKN1A* expressing cells as percentage of all cells in a cluster for IBM and NDC (Fig. 2d). As described in the previous section, the relative frequencies of *CDKN1A*^+^ nuclei were comparable between IBM and NDC for myonuclei. Intriguingly, the frequencies of *CDKN1A* expressing FAPs and monocytes/macrophages were strongly increased in IBM compared to NDC. However, it should be noted that the expression of a single gene is unlikely to capture the heterogeneity of cellular senescence across tissues. To address this caveat and to further corroborate the engagement of senescence pathways, we determined the differentially expressed genes (DEGs) between IBM and NDC for all cell clusters. Next, we employed the SenMayo gene set for enrichment analysis [56]. Briefly, the SenMayo gene set is a panel composed of 125 key genes associated with senescence signalling pathways. We chose SenMayo as this gene set has been benchmarked against existing senescence or SASP gene sets and outperformed the latter in the detection of senescent cells [56]. DEG lists of all clusters were entered into GSEA for the SenMayo gene set (Fig. 2e). Only the FAPs cell cluster was enriched for SenMayo in IBM compared to NDC. SnRNA-seq demonstrates the capability of discerning distinct cellular subtypes and suggests that non-myogenic cells assume a senescent phenotype in IBM.

### Single-nuclei RNA sequencing identifies a novel population of senescent FAPs that resides in IBM muscle

**Fig. 3:**
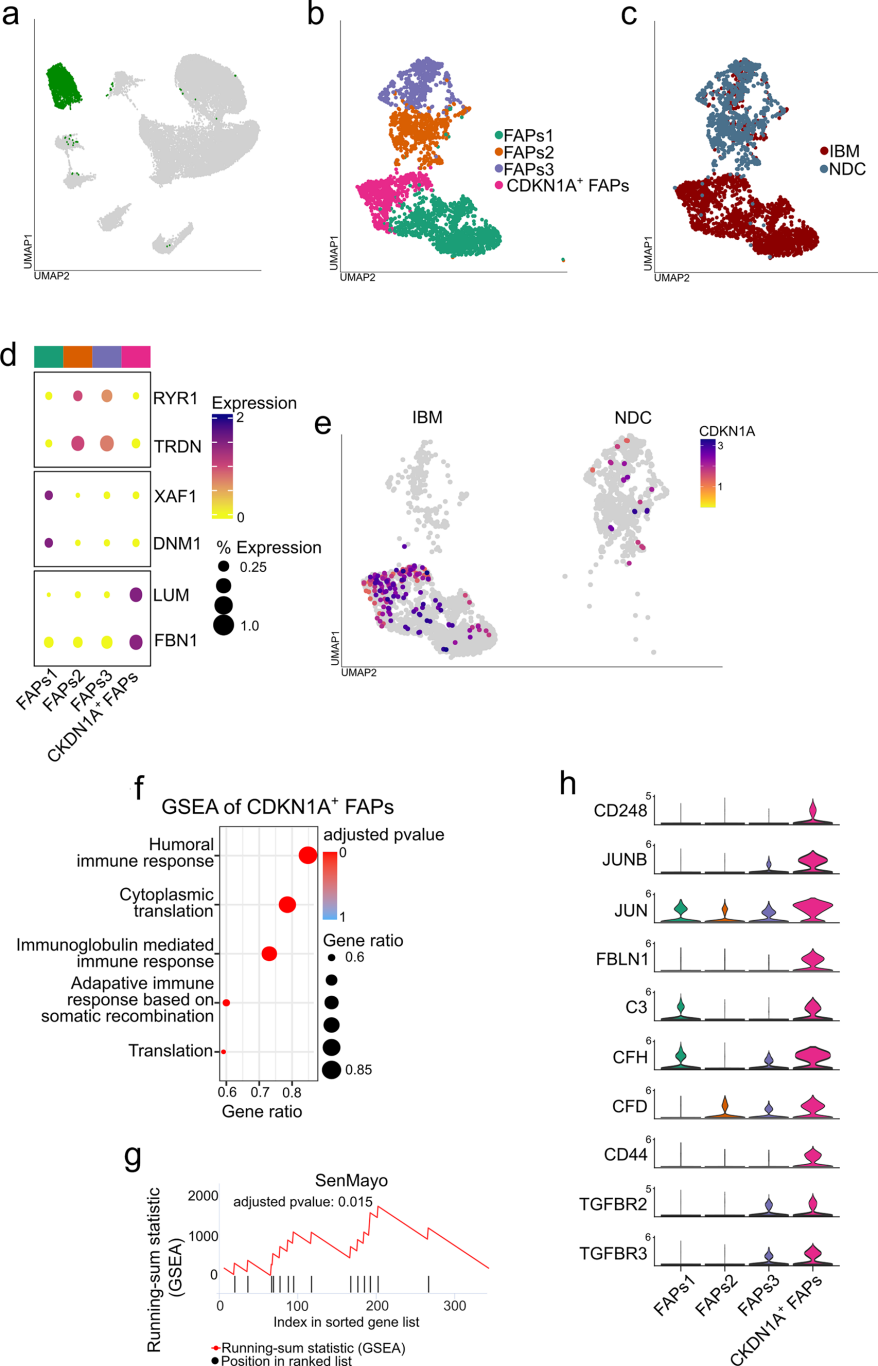
A novel population of senescent FAPs resides in IBM muscle. **a** UMAP embedding of the full dataset. FAPs are highlighted in green. All FAPs were extracted for downstream analysis and subclustering. **b** Subcluster analysis of the FAP population. Four FAP populations are identified based on their marker genes. **c** UMAP embedding displaying the origin for each nucleus. **d** Clustered dot plot visualization of top-regulated marker genes. The mean expression for each cluster is indicated by colour code. The dot size indicates the percent of expressing cells. Cluster were annotated based on marker genes. **e** Expression of CKDN1A (coding for p21) across the UMAP embedding split into the IBM (left) and NDC (right) datasets. The mean expression for each cell is indicated by the colour code. **f** Gene set enrichment analysis (GSEA) for the GO-BP dataset for the CDKN1A^+^ FAP cluster. Differentially expressed genes were determined by the FindMarkers function using Wilcox testing and a fold-change threshold of 0.25. The Bonferroni correction was used for correction for multiple testing. DEGs specific to the IBM dataset were entered into the GSEA. The Kolmogorov–Smirnov test, followed by a post hoc correction, was used to determine the significance. **g** GSEA analysis for the SenMayo dataset for the DEGs obtained from the CDKN1A^+^ FAP cluster. The running-sum statistic is in red, with the position in the ranked DEG list in black. Genes were sorted by fold change. The Kolmogorov–Smirnov test, followed by a post hoc correction, was used to determine the significance. **h** Violin plots displaying the normalized gene expression of the indicated genes for each FAP cluster. *CDKN1A* cyclin dependent kinase inhibitor 1A; *DNM1* dynamin-1; *FBN1* fibrillin-1; *GO-BP* gene ontology biological processes; *LUM* lumican; *NDC* non-diseased control; *IBM* inclusion body myositis; *RYR1* ryanodine receptor 1; *TRDN* triadin; *UMAP* uniform manifold approximation and projection; *XAF1* XIAP-associated factor 1

Next, we focused on the FAP population and extracted this cell cluster for in-depth analysis (Fig. 3a). A total of ~ 3200 FAP nuclei were analysed. FAPs are the lineage precursors of specialized non-myogenic cells, including activated fibroblasts, adipocytes, and osteogenic cells [8]. We performed subclustering on these cells and observed 4 subpopulations of FAPs (Fig. 3b, c). We determined marker genes for all FAP subtypes and compared these with previous studies [9, 55]. Although there currently exists no consensus regarding canonical marker genes of human FAPs, Perez et al. previously reported subtypes of FAPs associated with ageing based on the expression of the ryanodine receptor 1 (*RYR1*) and triadin (*TRDN)* [50]. In our dataset, the clusters FAPs2 and FAPs3 were also characterized by *RYR1* and *TRDN* expression. Given the advanced age of IBM patients and NDCs in our cohort, we suspect that these FAPs are reminiscent of these previously reported FAP phenotypes. In contrast, we also determined two clusters of FAPs only found in IBM patients, but not in NDCs. The first cluster was defined by the expression of lumican (*LUM*) and fibrillin-1 (*FBN1*). LUM^+/^FBN1^+^ FAPs were previously described [55] and are thought to resemble neprilysin (*MME*) expressing FAPs associated with fatty infiltration of skeletal muscle. Intriguingly, we also investigated the expression of *CDKN1A* across FAP subtypes and found that the expression of this senescence marker is largely restricted to the LUM^+^/FBN^+^ FAP population. Consequently, we termed these FAPs CDKN1A^+^ to underline their senescent phenotype and association with the p21 pathway. This FAP phenotype is found in IBM but not in NDCs (Fig. 3e). The identity of the fourth FAP subtype (FAPs3) is characterized by the expression of XIAP-associated factor 1 (*XAF1*) and dynamin-1 (*DNM1*)*.* These markers were previously described in FAPs [19], however, their role is currently unknown.

Given the scope of this study, we focused our analysis on the CDKN1A^+^ FAP population. To understand whether these FAPs exhibit senescence features, we determined the marker genes for this cell cluster and performed GSEA for the biological processes (BP) database (Fig. 3f). Here, the humoral immune response was the most enriched term for the CDKN1A^+^ FAP population. This observation is consistent with current knowledge on the senescent phenotype, as these cells are known to engage a pro-inflammatory secretome (e.g. SASP). To further corroborate a senescent phenotype for these FAPs, we performed GSEA for the SenMayo gene set. Here, marker genes of CDKN1A^+^ FAPs were enriched for SenMayo indicating the engagement of senescence pathways in these cells (Fig. 3g). We manually screened the marker genes of CDKN1A^+^ FAPs and observed that these cells express endosialin (CD248), a glycoprotein and key regulator of tissue fibrosis [48, 51, 59]. CD248 expression was only detected in CDKN1A^+^ FAPs but not in other FAP subtypes (Fig. 3h). Besides CD248, CDKN1A^+^ FAPs also engage the Jun/JunB signalling pathway [37, 56]. This pathway has been reported to govern fibroblast senescence by inhibition of insulin growth factor-1 (IGF-1) [37]. Surprisingly, CDKN1A^+^ FAPs also demonstrate upregulation of complement factors such as complement factor 3 (*C3*), complement factor H (*CFH*) or complement factor D (*CFD*). In respect to signalling, CDKN1A^+^ FAPs express CD44, a pro-inflammatory cell-to-cell signalling receptor, as well as receptors for transforming growth factor beta (TGF-β, *TGFBR2*, and *TGFBR3).* To corroborate that the p21+ cells in the perimysium/endomysium are indeed the FAP phenotype detected in the transcriptomic dataset, we performed IF staining of IBM biopsy specimens (Suppl. Figure 1). Here, p21^+^ cells located in the perimysium were identified by the canonical FAP marker platelet-derived growth factor receptor α (PDGRA).

To further cross-validate the engagement of senescence in these cells, we additionally investigated the activity of the senescence-associated β-galactosidase (SA-β-Gal) [38, 60]. To enable intracellular staining in tissue sections, we employed SPiDER-β-Gal which exerts higher cell permeability than traditional SA-β-Gal [29]. Indeed, p21 (CDKN1A)^+^ FAPs were detected in the perimysium of IBM patients and stained positive for SA-β-Gal as an additional biomarker for cellular senescence (Suppl. Fig. 2a).

Succinctly, CDKN1A + FAPs demonstrate key features of cellular senescence including expression of senescence markers, engagement of a pro-inflammatory secretory phenotype and pro-fibrotic surface molecules, the activity of SA-β-gal and a senescence-associated transcriptomic signature.

### IBM demonstrates a pronounced loss of type 2A muscle fibers

**Fig. 4:**
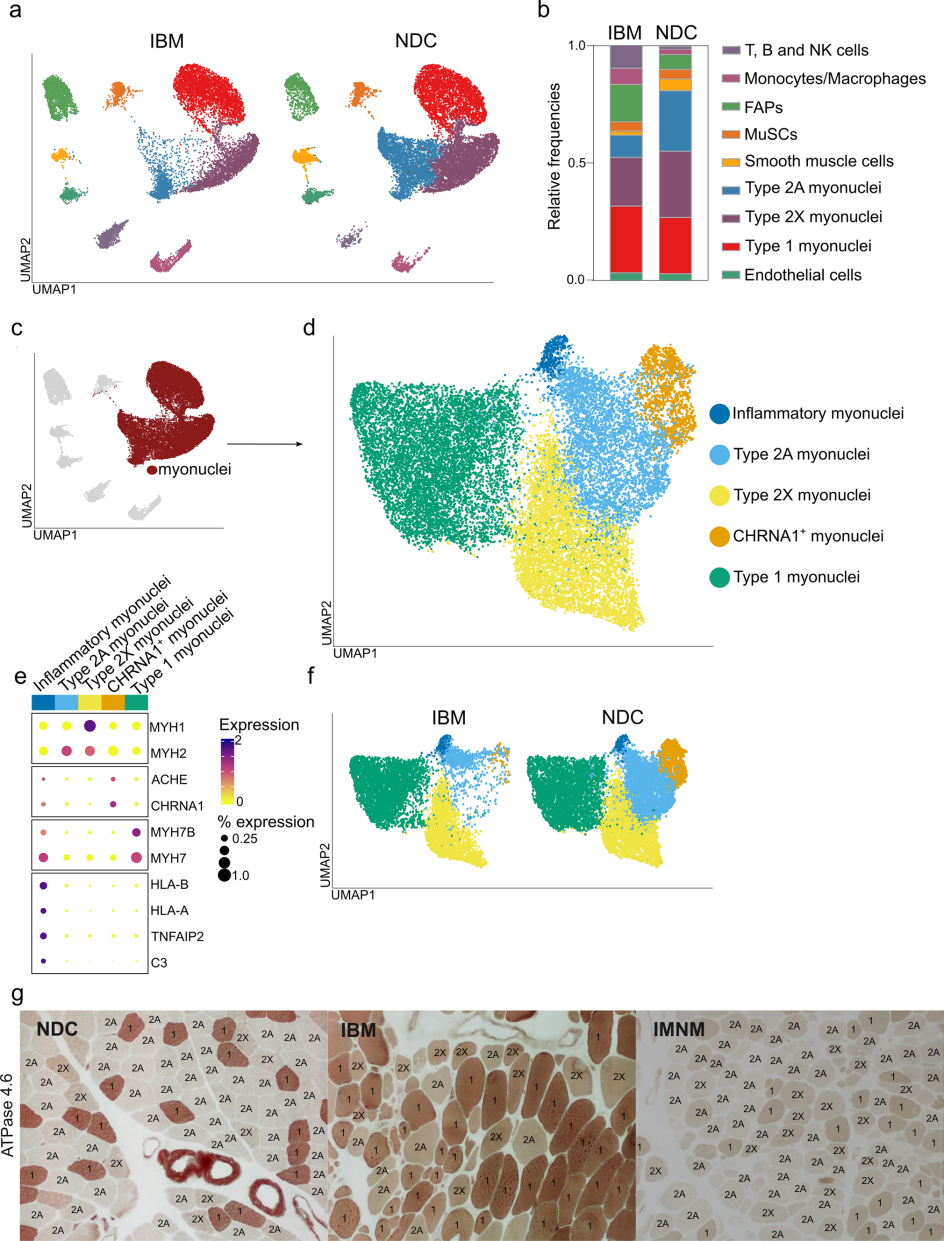
IBM demonstrates a pronounced loss of type 2A muscle fibers. **a** UMAP embedding of the full dataset split into IBM (left) and NDC (right). Cell populations are indicated by their colour code. **b** Relative frequency of each cell type or subtype in the IBM and the NDC dataset as a stacked bar plot. **c** UMAP embedding of the full dataset. Myonuclei are highlighted in dark red. These nuclei were extracted for downstream analysis **d** UMAP embedding of the myonuclei subclusters. A total of five populations were obtained from subclustering. **e** Clustered dot plot visualization of top-regulated marker genes. The mean expression for each cluster is indicated by colour code. The dot size indicates the percent of expressing cells. Clusters were annotated based on marker genes. **f** UMAP embedding for the myonuclei subcluster split into the IBM (left) and NDC (right) datasets. Subclusters are colour coded. **g** Exemplary ATPase staining for muscle specimens obtained from NDC, IBM, and IMNM patients. 8 patients were analysed by ATPase staining for each group. Muscle slices were incubated at a pH of 4.6, inactivating the myosin-ATPase of specific muscle fiber types. Type 1 muscle fibers are dark brown, type 2A is light brown, and type 2X are of an intermediate colour. The contrast and intensity vary between muscle specimens. A total of 10 high-power fields were counted. The corresponding statistical analysis is displayed in Suppl. Fig. 1B. *ACHE* acetylcholine esterase; *CHRNA1* cholinergic receptor nicotinic alpha 1 subunit; *FAP* fibro-adipogenic progenitor; *HLA* human leukocyte antigen; *IMNM* immune-mediated necrotizing myopathy; *MYH* myosin heavy chain; *MuSC* muscle stem cell; *NDC* non-diseased control; *IBM* inclusion body myositis; *TNFAIP2* TNF Alpha Induced Protein 2; *UMAP* uniform manifold approximation and projection

Next, we analysed the myonuclear compartment of IBM and compared it to NDCs (Fig. 4a). Consistent with the current knowledge on IBM histopathology [17], the immune cell clusters of monocytes/macrophages and T, B, and NK cells were expanded in IBM compared to NDC (Fig. 4a, b). Further, we also observed that the number of type 2A and to a lesser extent type 2X myofibers were strongly reduced in IBM compared to NDC.

To gain further insight into the phenotype of myogenic cells in IBM, we extracted the myonuclei from the full dataset (Fig. 4c) and performed subclustering on these nuclei (Fig. 4d). ~ 20,000 myonuclei were analysed by this approach. Subclustering revealed five distinct populations of myonuclei. Briefly, we detected type 1 myonuclei defined by *MYH7* and *MYH7B* expression as well as type 2X myonuclei expressing *MYH1* (Fig. 4e). Type 2A myonuclei were defined, as above, by the lack of *MYH1* and *MYH7* while expressing *MYH2*. This subclustering revealed two additional clusters of myonuclei. First, we detected a population of myonuclei that were defined by the expression of the acetylcholine receptor (*CHRNA1*), the acetylcholine esterase (*ACHE*) and the muscle associated receptor tyrosine kinase (*MUSK*). Given their unique expression profile of NMJ proteins, these nuclei are likely to belong to myonuclei constituting the neuromuscular synapse (NMJ). Second, a cluster of myonuclei emerged that expressed high levels of pro-inflammatory molecules, such as *HLA-A*, *HLA-B* or *HLA-C* constituting the major histocompatibility complex (MHC) class 1. Further, these myonuclei displayed TNF alpha-induced protein 2 (*TNFAIP2*) and complement protein 3 (*C3*) as marker genes. Given their unique expression profile of pro-inflammatory genes, we termed these myonuclei as inflammatory. Phenotypically, these myonuclei express *MYH7* and *MYH7B* suggesting that they resemble type 1 myofibers. Comparing IBM and NDC, there was a strong reduction in the number of type 2A myonuclei (Fig. 4f). Concurrently, CHRNA1^+^ myonuclei were also strongly reduced, while type 1 and type 2X myonuclei appeared largely unaltered.

For cross-validation of the specific loss of type 2A myofibers, we determined the frequency of myofiber types based on their ATPase staining after incubation at pH 4.6 [58]. We chose this approach to demonstrate the reduction in type 2A myofibers on the protein level and in a method independent of the MYH classification. Here, type 2A myofibers were markedly reduced in IBM compared to NDCs or IMNM patients (Fig. 4g, Suppl. Fig. 2b). In line, the relative frequency of type 1 myofibers was higher in IBM compared to the other groups. As such, a selective loss of type 2A myofibers appears to characterize the pathomorphology of IBM.

### Skeletal muscle cells assume an inflammatory reprogramming in IBM

**Fig. 5:**
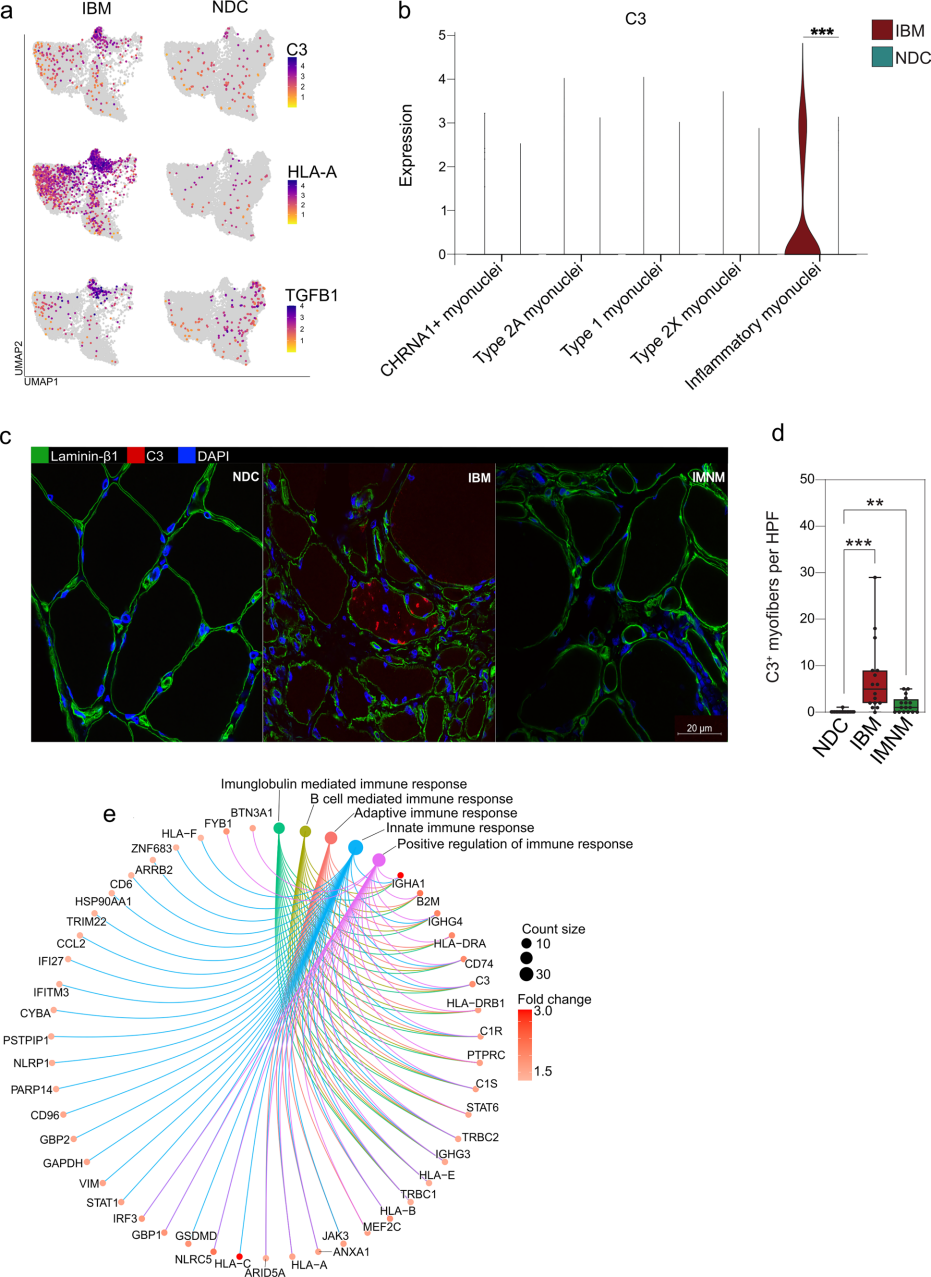
Skeletal muscle cells assume an inflammatory reprogramming in IBM. **a** UMAP embedding of the myonuclei split into IBM (left) and NDC (right). The expression of each gene indicated on the right is colour coded. **b** Violin plot displaying the expression of complement factor 3 (C3) for each myonuclei subset for IBM and NDC. Only inflammatory myonuclei obtained from IBM patients express C3. **c** Exemplary staining for C3 (red) laminin-β1 (green) and DAPI (blue) for NDC, IBM, and IMNM patients. 16 patients were analysed for each group by immunofluorescence. **d** C3^+^ myofibers were counted in randomly distributed 10 HPF (≙ 0.16 mm^2^). The biopsies were blinded for quantification, making the diagnosis impossible to identify from the label. **e** Visualization of the gene set enrichment analysis (GSEA) for the GO-BP dataset as a gene concept network. GSEA was performed from the DEGs comparing inflammatory myonuclei from IBM patients and NDC. The Kolmogorov–Smirnov test followed by post hoc correction was used to determine the significance. The top five GO terms for the BP dataset are colour coded. A line connects the corresponding genes constituting each term. The fold change for each gene comprising the GO term comparing IBM and NDC is indicated in red. The number of genes for each GO term is indicated as a dot size. *CHRNA1* cholinergic receptor nicotinic alpha 1 subunit; *HLA* human leukocyte antigen; *HPF* high-power field; *MYH* myosin heavy chain; *NDC* non-diseased control; *IBM* inclusion body myositis; *TGFB1* transforming growth factor beta 1; *UMAP* uniform manifold approximation and projection

Next, we focused on the subpopulation of inflammatory myonuclei. Intriguingly, while this population was detected in IBM and NDC, their phenotype was altered depending on their origin (Fig. 5a). These myonuclei demonstrated high expression of marker genes such as *C3* or *HLA-A* only in IBM, but not in NDC. Notably, these inflammatory myonuclei were also the primary source of transforming growth factor beta (*TGFB1*). Further, we sought to cross-validate the presence of these myonuclei in muscle. For this purpose, we performed IF staining for intracellular C3 as expression for this gene was only detected in inflammatory myonuclei from IBM patients, but not in other subtypes of myonuclei in either NDC or IBM suggesting sufficient specificity to serve as a marker (Fig. 5b). Indeed, myofibers with intracellular C3 staining were abundant in IBM compared to NDC or IMNM muscle (Fig. 5c, d). To further characterize the phenotype of these myofibers, we computed the DEGs comparing inflammatory myonuclei between IBM and NDC. GSEA analysis of these DEGs for the GO-BP database indicated engagement of pro-inflammatory pathways, including the immunoglobulin mediated immune response, adaptive immune response, innate immune response and, interestingly, the B cell mediated immune response (Fig. 5e).

### IBM myofibers lose their potential for endplate formation

**Fig. 6:**
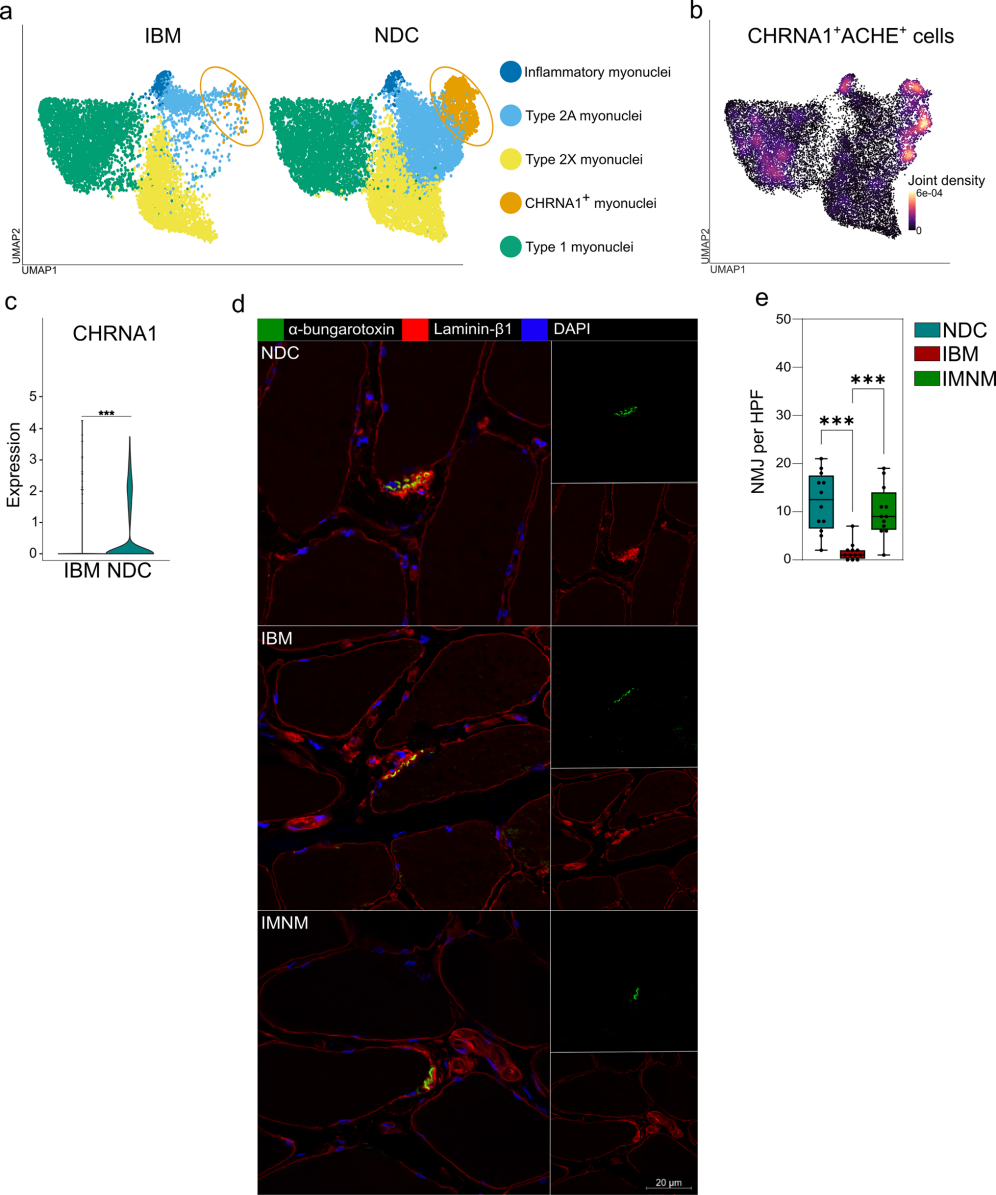
IBM myofibers lose their potential for endplate formation. **a** UMAP embedding of the myonuclei split into IBM (left) and NDC (right). The myonuclei cluster expressing the acetylcholine receptor (CHRNA1) is marked in orange. **b** Density plot generated with the Nebulosa package of cells expressing acetylcholine receptor and acetylcholine esterase (ACHE). Areas of high cellular density are indicated in orange. **c** Violin plot for the CHRNA1 expression across the dataset. CHRNA1-expressing nuclei are largely absent in IBM muscle. **d** Exemplary staining of a NMJ in NDC, IBM, and IMNM patients, respectively. α-bungarotoxin was used to label the acetylcholine receptors of the NMJ in green. 12 patients were analysed for each group. **e** While NMJ was detected in all muscle samples, their frequency was strongly reduced in IBM. NMJ was defined by its characteristic topography and expression of the acetylcholine receptor. NMJ was counted in randomly distributed 10 HPF (≙ 0.16 mm^2^). The biopsies were blinded for quantification, with the diagnosis impossible to identify from the label. 12 patients were analysed by immunofluorescence for each group. **f** Frequencies of NMJ in NDC, IBM, and IMNM patients. Differences between groups were analysed by Kruskal–Wallis test followed by post hoc testing. ***p < 0.001. *ACHE* acetylcholine esterase; *CHRNA1* cholinergic receptor nicotinic alpha 1 subunit; *HPF* high-power field; *NDC* non-diseased control; *IBM* inclusion body myositis; *IMNM* immune-mediated necrotizing myopathy; *UMAP* uniform manifold approximation and projection

To further characterize the changes to the myogenic compartment in IBM, we aimed to validate the reduction in AChR (*CHRNA1*) expressing muscle fibers (Fig. 6a). On the transcriptomic level, cells concurrently expressing the AChR as well as the acetylcholine esterase (*ACHE*) were reduced in IBM (Fig. 6b). Similarly, the expression of CHRNA1 in myonuclei was detected in NDC, but not in IBM (Fig. 6c). Formation of the NMJ is required for nerve-muscle communication. To visualize the neuromuscular endplate, we performed IF for α-bungarotoxin binding the AChR and counted the number of NMJs. Here, the number of NMJs was strongly reduced in IBM compared to NDCs or IMNM patients (Fig. 6d, e). NMJs were defined by the concurrent binding of α-bungarotoxin and their characteristic topography. These data suggest that endplate formation is impaired in IBM muscle potentially affecting nerve-muscle communication.

Conclusively, single nuclei analysis suggests that the myogenic compartment of IBM is characterized by the loss of type 2A myonuclei and of myonuclei that constitute the NMJ. Conversely, C3 expression identifies a distinct pro-inflammatory subpopulation of myonuclei residing in IBM muscle.

### FAPs demonstrate a shifted collagen homeostasis with potential consequences for muscle health in IBM

**Fig. 7:**
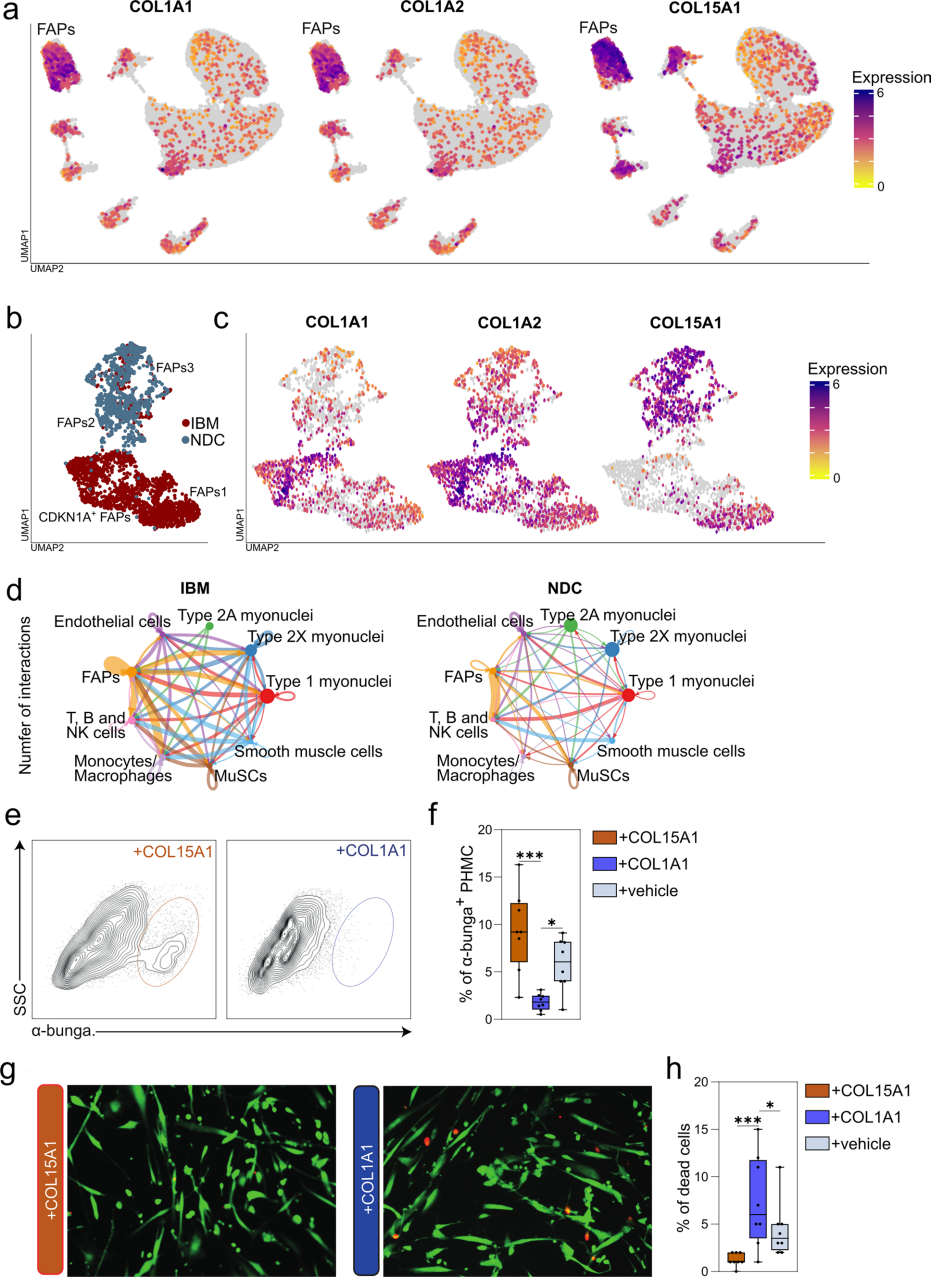
FAPs demonstrate a shifted collagen homeostasis with potential consequences for muscle health in IBM. **a** UMAP embedding of the full dataset with the corresponding gene expression indicated by the colour code. The FAP cluster is in the upper left. **b** UMAP of the FAP subclusters split into their origin of IBM patients or NDC. The annotation of the corresponding subcluster is given beside the plot. CDKN1A^+^ FAPs are in the lower left. **c** UMAPs demonstrating COL1A1, COL1A2, and COL15A1 expression across the FAP cluster and its subclusters. COL15A1 is largely absent in the CDKN1A^+^ FAP subcluster. **d** Analysis of the cell–cell communication across the IBM and NDC datasets using the CellChat package. The number of the inferred ligand/receptor interactions is indicated by the size of an arrow connecting two cell populations. The larger the arrow, the higher the number of interactions between the cell populations. The ligand/receptor pairings were tested against the CellChat library for significance. **e** Representative flow cytometry scatter plots displaying SSC vs α-bungarotoxin for primary human muscle cells (PHMC) treated with COL15A1 or COL1A1. **f** Percentage of α-bungarotoxin + PHMC treated with COL15A1 or COL1A1 or vehicle. *N* = 8 per group. **g** Representative live/dead staining for PHMCs incubated with COL15A1 or COL1A1 in addition to 10 ng/ml interferon-γ (INF-γ). Live cells were identified by green-fluorescent calcein-AM, indicating intracellular esterase activity. Dead cells were identified by red-fluorescent ethidium homodimer-1, indicating a loss of membrane integrity. **h** 100 cells were counted for each sample, and the frequencies of live or dead cells were recorded. *N* = 8 per group. Differences between groups were analysed by Kruskal–Wallis test followed by post hoc testing. *p < 0.05, ***p < 0.001. *CDKN1A* cyclin dependent kinase inhibitor 1A; *COL* collagen; *FAP* fibro-adipogenic progenitor; *NDC* non-diseased control; *IBM* inclusion body myositis; *IMNM* immune-mediated necrotizing myopathy; *UMAP* uniform manifold approximation and projection

Finally, we aimed to determine whether senescence of FAPs may be linked to changes to the myogenic compartment. FAPs and their fibroblast lineage are the primary sources of extracellular matrix proteins required for skeletal muscle homeostasis [6]. Manual screening of the top dysregulated genes in CDKN1A^+^ FAPs revealed a shift in the expression of collagens, specifically of COL15A1, an isoform coding for collagen type XV, as well as COL1A1 and COL1A2, coding for collagen type I. Expression of all three collagen coding genes is allocated to the FAP cluster in both NDC and IBM (Fig. 7a). However, further analysis of the FAP subcluster indicates a shift of collagen expression between IBM and NDCs with CDKN1A^+^ FAPs demonstrating a sharp downregulation of COL15A1 (*p* = 5 × 10^–32^), while engaging COL1A1 (*p* = 1 × 10^–87^) as well as COL1A2 (*p* = 1 × 10^–70^) expression (Fig. 7b, c). Both types of collagens are required to stabilize skeletal muscle cells and their differentiation. However, they vary in their biological functions [6, 24].

Following this line of argumentation, we suspected that differences in collagen might affect skeletal muscle cell homeostasis. To understand the communication between FAPs and myofibers better, we analyzed cell–cell communication across the transcriptomic dataset. We used the CellChat package to calculate the inferred intercellular communication based on an established receptor/ligand database [27]. We extracted all nuclei acquired from IBM patients and NDCs for individual analysis. Here, quantification of significantly over-expressed ligand/receptor pairings revealed that cell–cell communication is amplified across cell types and subtypes in IBM compared to NDCs (Fig. 7d). Intriguingly, FAPs appear to be a major communication hub interacting with myonuclei, immune cells and MuSCs. Indeed, the collagen and laminin signalling pathways constituted most outgoing ligands in IBM FAPs. Both signalling pathways belong to the KEGG pathway of extracellular matrix-receptor interaction (hsa04512). We suspect that a shift of the extracellular matrix composition might have functional implications for myofiber health in IBM.

To test this assumption in vitro, we cultivated primary human muscle cells (PHMC) and analysed them by flow cytometry. After reaching full confluence, PHMCs were treated with either 20 μg/ml of COL15A1 or COL1A1 [26, 33]. As specific collagen compositions are required for the successful maturation of the NMJ [33, 47], we analysed the amount of NMJ forming myofibers by quantification of α-bungarotoxin (Fig. 7e). In this setup, treatment with recombinant COL15A1 or with vehicle improved NMJ formation as compared to COL1A1. Next, we also aimed to understand if differences in collagen composition might affect PHMC survival. For this purpose, differentiated PHMCs were treated with 20 μg/ml of COL15A1 or 20 μg/ml COL1A1. To reflect inflammatory conditions in IBM, PHMCs were additionally treated with 10 ng/ml interferon-γ (INF-γ). After 48 h of incubation, we determined the number of dead PHMCs by live/dead staining (Fig. 7g, h). Interestingly, COL15A1 improved PHMC survival in response to INF-γ compared to COL1A1 or vehicle. These data suggest that senescent FAPs demonstrate an altered collagen homeostasis shifted towards collagen type I.

## Discussion

FAPs are a mesenchymal cell population with high phenotypical plasticity that is crucially involved in skeletal muscle homeostasis and regeneration [5, 23]. Here, we report that tissue-resident FAPs, not myofibers, are the main cell type assuming a senescent phenotype in IBM. Depending on environmental cues, FAPs may differentiate into fibroblasts or adipocytes. In response to muscle damage, FAPs proliferate, expand and accumulate, constituting the main source of extracellular matrix proteins [23, 41]. Depletion of FAPs hinders muscle repair underscoring their functional importance [23, 41]. Conversely, in conditions of chronic muscle damage, FAPs may prove detrimental to muscle health. Their persistent activity cumulates in progressive tissue fibrosis and loss of normal tissue architecture [22, 23]. In line, we describe a novel population of senescent FAPs that reside in IBM muscle. These FAPs exhibit key hallmarks of cellular senescence including a pro-inflammatory secretome, engagement of the Jun/JunB-pathway and expression of senescence biomarkers (p21 and SA-β-Gal).

Besides their inflammatory properties, these FAPs also exhibit a shift in their collagen balance losing collagen type XV and increasing collagen type I expression. Interestingly, Col15a1 knockout mice lacking collagen type XV demonstrate a myopathy vulnerable to exercise-induced muscle injury, suggesting that this collagen type is required for muscle homeostasis [11]. Histopathologically, this myopathy is characterized by areas of degeneration and regeneration as well as variations in myofiber size [11]. Unlike other collagen types, collagen type XV is only encoded by a single gene (COL15A1) [25]. As such, a loss of COL15A1 expression, as seen in CDKN1A^+^ FAPs, will likely result in impaired collagen type XV production. Collagen interacts with skeletal muscle via a number of receptors, such as integrins, mediating a plethora of downstream effects [63]. Besides differentiation and regeneration, collagen-muscle cell interaction is also required for the maturation and formation of the NMJ. Animal studies demonstrated that collagen type XIII and type XIX are needed for successful NMJ genesis [33, 47]. While the exact molecular pathways remain to be studied, we hypothesize that collagen dysregulation and persistent pro-inflammatory activity could link FAP senescence and the loss of structural integrity of the muscle compartment in IBM. Current immunosuppressive approaches are unlikely to inhibit or even affect the persistent activity of FAPs. Following this line of argumentation, senescent FAPs may represent a cell-autonomous mechanism that sustains inflammation and fibrotic remodelling in IBM despite therapeutically addressing immune cell pathology warranting further research.

Expanding on previous proteomics data that demonstrated a loss of type 2 myofibers linked to impaired glycolysis in IBM [49], snRNA-seq provides further insight into changes to the myogenic compartment in IBM at a high resolution. Here, the loss of type 2 fibers allocates specifically to the subtype of type 2A myofibers. Further, the transcriptomic landscape of IBM displays a rarefication of neuromuscular endplates and the engagement of an inflammatory muscle phenotype. Differences in composition and metabolic demands render individual myofibers vulnerable to distinct conditions. As such, type 2A myofibers rely primarily on oxidative phosphorylation for their ATP supply, while type 2X myofibers have an effective glycolytic ATP production [58]. Mitochondrial dysfunction is a primary feature of IBM [46], thus providing a potential explanation for the pattern of myofiber loss observed. We hypothesize that type 2A myofibers are unable to meet their metabolic demand due to mitochondrial dysfunction in IBM, while type 2X myofibers may rely on glycolytic ATP. Further research on muscle metabolism and immunogenicity might prove valuable to better understand this understudied aspect of IBM pathophysiology. The importance of the myogenic compartment is further stressed by the possibility of denervation contributing to pathology. Clinical, histomorphological and electrophysiological data suggest a neurogenic component and a failure of NMJ transmission in IBM [17, 45]. Here, we provide molecular evidence in favour of this hypothesis as myofibers constituting the NMJ appear to decline in IBM. Further research is warranted to understand this aspect of pathomorphology and whether treatment strategies addressing the NMJ, such as pyridostigmine, could prove valuable for the clinical management of IBM.

A recurrent observation across the transcriptomic dataset was the dysregulation of intracellular complement components. Historically, complement has been viewed as a serum-effective system that mediates the detection and removal of invading pathogens [62]. Intriguingly, recent studies challenged this classical view by demonstrating that complement activation not only occurs on the cell surface, but also in intracellular compartments, thereby regulating cell physiology of immune and non-immune cells [3, 31, 62]. In this study, we observed that inflammatory states of both myogenic and non-myogenic cells were accompanied by the upregulation of complement proteins, particularly of C3. Indeed, intracellular complement proteins are coded by the same set of genes that give rise to the circulatory complement system [62]. Moreover, intracellular complement regulates tissue inflammation and the transcriptomic programming of fibroblasts [13]. In response to repeated inflammatory challenges, fibroblasts in humans and mice assume a ‘primed’ phenotype that renders tissue susceptible to sustained inflammation. This transcriptomic program is controlled by C3 and the C3a-receptor as well as the mammalian target of rapamycin (mTOR). Following this line of argumentation, we suspect that the engagement of complement factors in this transcriptomic dataset suggests altered intracellular complement signalling as a potential contributor to IBM pathophysiology warranting further research.

Concurrently, an interesting argument for cellular senescence might arise from the effect of sirolimus in IBM. In a recent randomized, placebo-controlled trial for IBM, the mTOR inhibitor sirolimus did not meet the primary outcome of maximal voluntary isometric knee extension strength; however, secondary outcome measures such as 6-min walking distance, forced vital capacity and thigh fat fraction were ameliorated [4]. Aside from immunoregulatory effects, mTOR signalling has been linked to cellular senescence. In vivo and in vitro evidence demonstrated that the mTOR pathway suppresses the SASP, e.g. via Mitogen-activated protein kinase 2 (MAPK) signalling, of senescent cells [20, 32]. Hence, sirolimus is likely to inhibit pro-inflammatory fibroblasts requiring mTOR signalling, as described above [13]. Thus, it is tempting to speculate that the therapeutic effect of sirolimus is—at least in part—mediated by modulation of the senescent fibroblasts.

This study employs snRNA-seq as opposed to scRNA-seq. Both methodological approaches offer specific advantages and disadvantages. In respect to myofibers, the large size of individual cells limits the use of scRNA-seq rendering snRNA-seq a preferential choice [9, 50]. Concurrently, snRNA-seq also allows for the analysis of frozen tissues, thereby enabling the collection and analysis in a single run reducing the risk for batch effects [30]. A limitation of this approach is the complexity of multinucleated cells, as we cannot determine if certain nuclei originated from the same myofiber. Further, depending on the chosen method [59], specific cell populations might be underrepresented. In the study of liver tissue, snRNA-seq detected a lower number of immune cells than scRNA-seq [61]. Combining snRNA-seq with scRNA-seq might provide a more comprehensive picture of the muscle compartment than an individual method alone. Finally, a potential limitation to this study is introduced by the focus on the distal segment of the quadriceps muscle. The highest concentration of NMJs is located within the equatorial region of the muscle [28]. This topographic disparity should be considered when interpreting these findings as further research is warranted to understand whether the reduction of NMJs in IBM patients is also present in the high-density equatorial segment of the muscle.

Taken together, this study highlights the contribution of dysfunctional tissue-resident cells to IBM pathophysiology. Treatment strategies for IBM may benefit from exploring an integrative approach that targets invading immune cells while also addressing detrimental tissue-resident cells such as FAPs and their senescent phenotype.

### Supplementary Information

Below is the link to the electronic supplementary material.

Supplementary file1 (XLSX 383 KB)

Supplementary file2 **a** Representative immunofluorescence staining of IBM muscle specimen. Muscle slices were incubated with SPiDER-βGAL at a pH of 6. SPiDER-βGAL indicates the activity of the senescence-associated β-galactosidase. SPiDER-βGAL stains green. Senescent FAPs were identified in the perimysium by p21 staining in red. **b** Quantification of myofiber types by ATPase 4.6 staining. An exemplary set of staining is given in Fig. 4. A total of 100 myofibers were counted for each sample. *N* = 8 per group. Differences between groups were analysed by Kruskal–Wallis test followed by post hoc testing. *p < 0.05, ***p < 0.001. *NDC* non-diseased control; *IBM* inclusion body myositis; *IMNM* immune-mediated necrotizing myopathy (TIFF 2509 KB)

Supplementary file3 **a** Representative immunofluorescence staining of IBM muscle specimen. FAPs were detected in the perimysium, and their phenotype was confirmed by staining for p21 (red), PDGFRA (green), CD248 (brown), and DAPI (blue). Here, two senescent FAPs are seen between two muscle fibers. *IBM* inclusion body myositis; *PDGFRA* platelet-derived growth factor receptor alpha (TIFF 2494 KB)

## Data Availability

The raw transcriptomic data are publicly available under the accession number: SCP2253 at https://singlecell.broadinstitute.org/single_cell. All further data are available from the corresponding author upon reasonable request.
