# Supplementary figures and images for: Senescent fibro-adipogenic progenitors are potential drivers of pathology in inclusion body myositis
Source: Acta Neuropathol. 2023 Sep 29;146(5):725–45. doi: 10.1007/s00401-023-02637-2 (PMC10564677; doi:10.1007/s00401-023-02637-2)

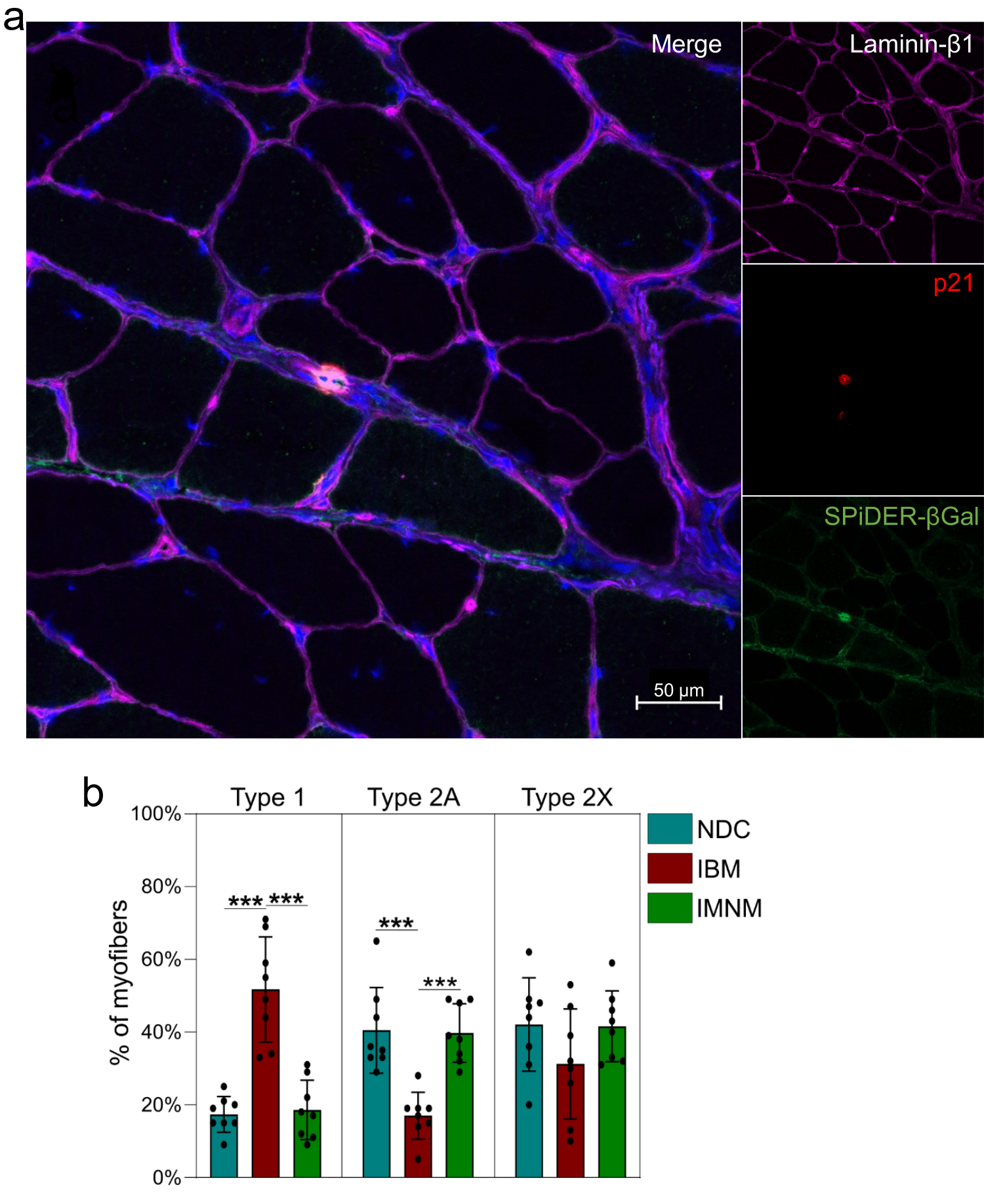

Supplement: Supplementary file 2 — Supplementary file2 a Representative immunofluorescence staining of IBM muscle specimen. Muscle slices were incubated with SPiDER-βGAL at a pH of 6. SPiDER-βGAL indicates the activity of the senescence-associated β-galactosidase. SPiDER-βGAL stains green. Senescent FAPs were identified in the perimysium by p21 staining in red. b Quantification of myofiber types by ATPase 4.6 staining. An exemplary set of staining is given in Fig. 4. A total of 100 myofibers were counted for each sample. N = 8 per group. Differences between groups were analysed by Kruskal–Wallis test followed by post hoc testing. *p < 0.05, ***p < 0.001. NDC non-diseased control; IBM inclusion body myositis; IMNM immune-mediated necrotizing myopathy (TIFF 2509 KB) [file 401_2023_2637_MOESM2_ESM.tiff]

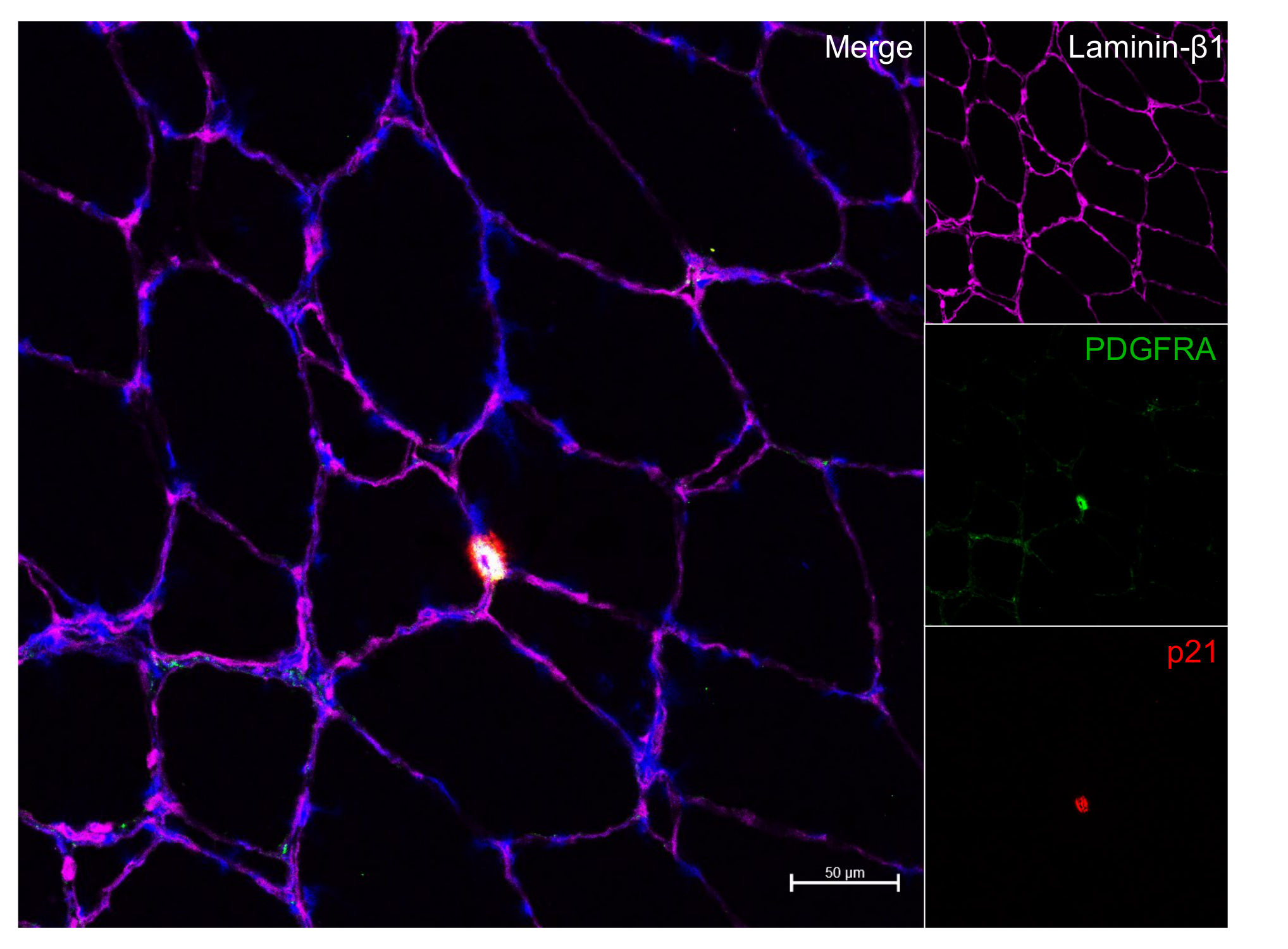

Supplement: Supplementary file 3 — Supplementary file3 a Representative immunofluorescence staining of IBM muscle specimen. FAPs were detected in the perimysium, and their phenotype was confirmed by staining for p21 (red), PDGFRA (green), CD248 (brown), and DAPI (blue). Here, two senescent FAPs are seen between two muscle fibers. IBM inclusion body myositis; PDGFRA platelet-derived growth factor receptor alpha (TIFF 2494 KB) [file 401_2023_2637_MOESM3_ESM.tiff]
